# Journey From a Digital Innovation to a Sustainable Health Worker Capacity-Building App in India: Experiences, Challenges, and Lessons Learned

**DOI:** 10.9745/GHSP-D-24-00006

**Published:** 2024-06-27

**Authors:** Rebecca Chase, Sohini Sanyal, Preksha Singh, Sharda Sharda, Anita Bhargava, Pramod Raturi, Gopal Krishna Soni, Parthasarathi Ganguly

**Affiliations:** a JSI Research & Training Institute, Inc., Arlington, Virginia, USA.; b John Snow India Private Limited, New Delhi, India.; c CottonConnect, Gurgaon, India; formerly of John Snow Inc India Private Limited.; d Parul Institute of Public Health, Parul University, Vadodara, India; formerly of John Snow Inc India Private Limited.

## Abstract

Health workers, especially auxiliary nurse midwives (ANMs), are among the most critical resources in improving the quality of immunization services and reducing vaccine hesitancy under the Universal Immunization Programme (UIP) in India. To improve health worker immunization skills, UIP trainings in India are primarily conducted through instructor-led classroom, cascade trainings. However, a 2018 capacity-building need assessment revealed several challenges involved in traditional classroom training, such as a single-time exposure to new guidelines, complicated logistics arrangements, a lack of refresher training, and varying quality of training. These complexities make it difficult to meet the timely knowledge and skill needs of every health worker effectively and uniformly in a rapidly changing scenario of UIP. To meet health worker capacity-building needs and address these challenges, Rapid Immunization Skill Enhancement (RISE), a learning management system (LMS) application, was conceptualized. The RISE LMS application was developed as a human-centered, interactive, continuous, and adaptable knowledge and skill-building platform for health workers engaged in the UIP. RISE complements existing classroom-based cascade training for health workers by leveraging digital technologies for faster, easier, and more effective knowledge transfer to accommodate the fast-changing needs of a dynamic health program like UIP. In this article, we share the challenges and strategic solutions to digital training applications, lessons learned, sustainability of the application, and the impact RISE has made in India, all of which stemmed from leadership, coordinated efforts from a team of skilled professionals, government acceptance, detailed planning, and continued stakeholder engagement.

## Abstract (precis)

We describe the development and implementation of a digital human-centered, interactive knowledge and skill-building platform to complement traditional classroom training and help meet the timely knowledge and skill needs of every health worker effectively and uniformly.

## Teaser

**Key Findings:**

- To train health workers on delivering immunization services, a traditional instructor-led classroom training approach posed several challenges, including variations in the quality of training, need for complicated logistics arrangements to implement, and lack of ability to provide refresher training.
- A digital platform was developed to complement classroom training and provide an interactive, engaging, faster, and more effective way to address gaps in health worker knowledge and skills in providing immunization services.
- After successfully piloting the app among 2,800 users in 5 districts in 5 states, the app has been scaled up to 40,000 users in 101 districts in 6 states in India, with plans for further scale-up across the country.

**Key Implication:**

- Digital learning platforms can facilitate faster and more effective knowledge and skill transfer and provide mechanisms for supportive supervision, peer-to-peer learning, and dissemination of national policy guideline updates.

## BACKGROUND

Immunization is one of the most cost-effective public health interventions, described as the foundation of a healthy, productive population by the World Health Organization.^1^ Vaccines are pivotal in providing important public health benefits in terms of avoiding deaths from vaccine-preventable diseases, increasing life-years saved, affecting disability-adjusted life years or quality-adjusted life years,^2^ and providing financial protection by preventing expenditure incurred by households due to vaccine-preventable illnesses.^3^ The Universal Immunization Programme (UIP) in India is one of the largest public health programs in the world^4^^–^^6^ under which all pregnant women and children can receive free vaccination services at the nearest government health facility or immunization post (i.e., anganwadi centers or other identified sites).^7^

Health workers (HWs), especially auxiliary nurse midwives (ANMs), are among the most critical resources in improving the quality of immunization services and reducing vaccine hesitancy under UIP.^8^^,^^9^ ANMs are based and provide care at a subcenter and perform a broad set of responsibilities, including supporting and training anganwadi workers and accredited social health activists.^4^^,^^10^^,^^11^ ANMs have been appointed as multipurpose workers who are required to provide child health and primary care services to communities, including family planning, immunization, infectious disease prevention and care, pregnancy and childbirth care, and mobilizing eligible families for sterilization.^4^^,^^10^^,^^11^ Therefore, ensuring ANMs are adequately qualified and trained becomes a key prerequisite in providing immunization services to communities.^8^ ANMs in the UIP receive training through mostly instructor-led classroom cascade training sessions. In 2018, a capacity-building need assessment in 5 states in India conducted by John Snow Inc India Pvt. Ltd. (JSIPL) reflected that using this training approach posed several challenges, including only providing a single-time exposure to new guidelines, using complicated logistics arrangements, lacking structured provision of refresher training, and providing varying quality of training.^12^ These complexities made it difficult to provide timely knowledge and skills to every HW effectively and uniformly in a rapidly changing scenario of UIP; thus, capacity-building was uneven and challenging. The capacity-building needs assessment included a technology scoping exercise that also indicated that more than 80% of the HWs possessed smartphones and, of that group, 84% were conversant with its use.^8^

To address this gap, JSIPL, a partner of the Government of India Ministry of Health & Family Welfare (MOHFW), conceptualized Rapid Immunization Skill Enhancement (RISE) (https://risemohfw.in/),^13^ a learning management system (LMS) application, as a training model for faster but effective knowledge and skill transfer to HWs to complement the existing classroom-based training and accommodate the fast-changing needs of a dynamic health program like UIP.^10^ The technology scoping exercise solidified that mobile phones were the best mode for training, given the already high digital literacy.^12^ RISE is an innovative, interactive, continuous, adaptable, blended, self-learning, and skill-building digital platform to assist HWs involved in the UIP in regularly updating their knowledge and skills.^13^ Supplement 1 presents the back-end technical specifications for the RISE LMS app. With the Government of India’s increased emphasis on extensive deployment of digital tools to improve the efficiency and outcome of the health care systems^3^^,^^14^^–^^16^ and the “Digital India” drive—the Government of India’s program to “transform India into a digitally empowered society and knowledge economy,”^17^ the RISE LMS was the first large-scale capacity-building app developed and used in the health sector in the country.

RISE was conceptualized as a training model for faster but effective knowledge and skill transfer to HWs to complement the existing classroom-based training and accommodate the fast-changing needs of a dynamic health program like UIP.

## RISE DESIGN AND DEVELOPMENT PROCESS

### Develop app content

The extensive capacity-building needs assessment involved 119 stakeholders from the national level (e.g., district collectors), state (e.g., state immunization officers, cold chain officers, information education and communication officers), district (e.g., district immunization officers, cold chain technology, information, education, and communication officers), block (block medical officers), and field levels.^12^ After the needs assessment, the MOHFW established a National Working Group to further identify areas of critical gaps in HWs’ knowledge and practice related to the open vial policy, vaccine schedule, waste management, and adverse events following immunization. The working group identified the following 5 core content topics to include as modules in the RISE LMS app: (1) Immunization Schedule and Session Management, (2) Injection Safety and Vaccine Administration, (3) Principles of Cold Chain Management, (4) Adverse Events Following Immunization, and (5) Communication to Tackle Vaccine Hesitancy.

Several JSIPL colleagues and MOHFW officials were responsible for the conceptualization of the main features of the RISE app. An external information technology agency was then onboarded to develop the LMS application. A 2-pronged approach was used to build the RISE content. First, training methodologies (e.g., adult learning principles, instructional design, and different learning styles [V-visual/A-aural/R-read/K- kinesthetic learners]) were used to make the content delivery innovative and impactful. Second, the content was presented in a lucid style and relatable context for ANMs, the RISE apps’ target audience. A familiar narrator figure was created representing a traditional medical officer, who is usually a direct supervisor to ANMs, to guide learners through the content. The content was presented in a lucid style free of jargon, presented in story format rather than as an instructional course, and in the learner’s primary language. In addition, the module backgrounds were made relatable by representing places where ANMs usually work (e.g., vaccination session sites) or interact with the community.

**FIGURE 1:**
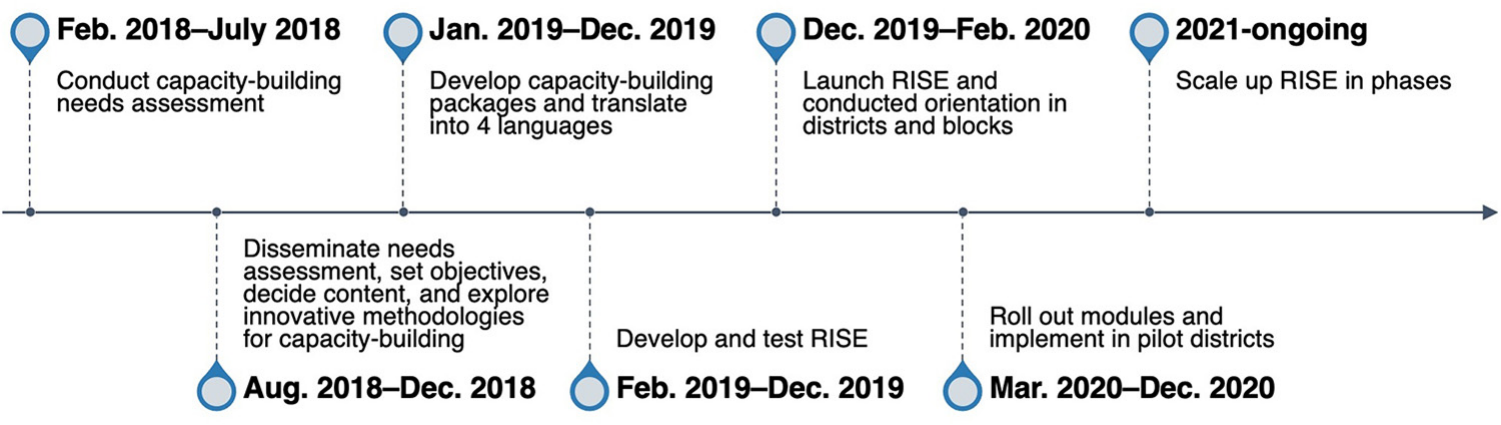
Timeline of Conceptualization and Implementation of the RISE Learning Management System Application

To keep learners engaged and help with knowledge recall, quizzes and games were embedded into the modules, and pre- and post-tests were developed as assessments. After the successful completion of each module assessment, HWs received a silver (for scoring 70%–85%) or gold (for scoring 86% or higher) certificate generated by the app, which provided them with motivation. Learners scoring 69% or lower had to reattempt the assessment. After completing all modules, learners received a master certificate. Real-time dashboards allowed supervisors to see the learning progress and completion of modules of colleagues under their jurisdiction and take targeted corrective actions if needed. The app was also translated and produced in Hindi, Marathi, Odia, and Tamil to accommodate non-English language learners.

### Pilot-Testing

Starting on March 1, 2020, the 5 modules of RISE were piloted to more than 2,800 users in 5 districts of 5 states: Shimla (Himachal Pradesh), Bhopal (Madhya Pradesh), Khordha (Odisha), Pune (Maharashtra), and Kancheepuram (Tamil Nadu) (Figure 1). At baseline and endline of the pilot phase, a knowledge assessment survey and a practice improvement assessment were conducted among ANMs in pilot districts and 5 control districts.^18^ The control districts were Solan (Himachal Pradesh), Indore (Madhya Pradesh), Jharsuguda (Odisha), Nagpur (Maharashtra), and Madurai (Tamil Nadu). In the control districts, it is unknown if any classroom-based trainings took place due to the COVID-19 pandemic lockdown. For the knowledge assessment, a multistage cluster sampling method was used to determine sample size. First, cluster sampling was used to identify blocks in 3 clusters (i.e., urban, peri-urban or urban hinterlands, and rural) in each intervention and control district. In end-stage sampling, simple random sampling was used to select 276 ANMs in 14 clusters of the 5 intervention districts and 258 ANMs in 14 clusters of the 5 control districts. Supplement 2 presents the sampling frame and size used in the intervention and control districts, and knowledge assessment survey findings.

**FIGURE 2:**
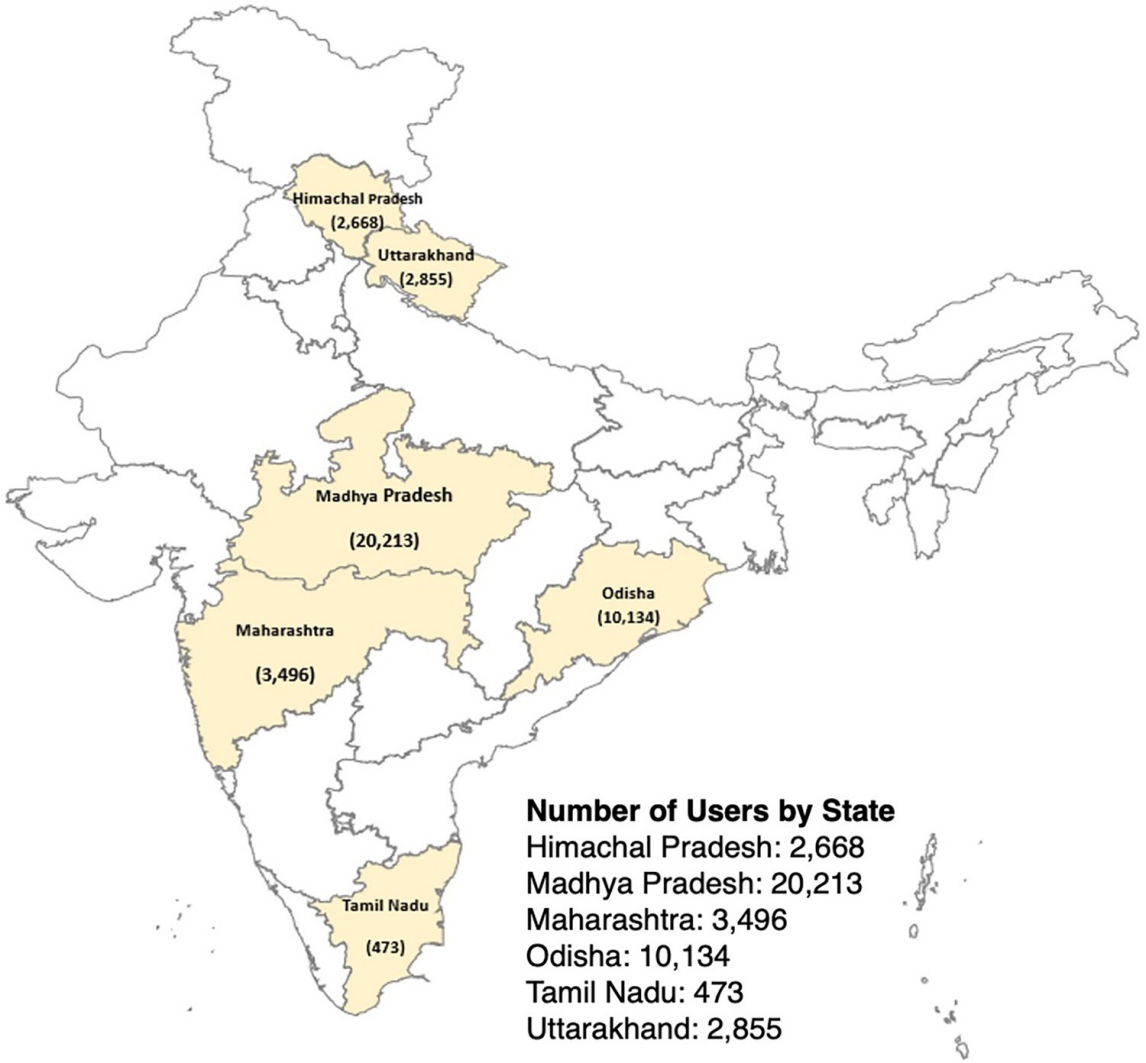
Map of RISE App Users in India, May 2024

In both the control districts and the intervention districts, the knowledge assessment survey questionnaire consisted of 30 questions, representing the 5 modules of RISE.^18^ JSIPL collected both the baseline and endline data using digital formats. Baseline data were collected from September to November 2019, and endline data from October to December 2020. The comparison between the baseline and endline knowledge assessment survey of the learners elucidates that there was a significant knowledge gain among the learners after taking the RISE modules. There was an observed gain of 14 points compared to the control districts observed at 5 points (Supplement 2).

After ANMs used the RISE app, an endline practice improvement assessment questionnaire was administered, comprising 11 key parameters assessing the practices of the UIP guidelines that ANMs performed at the session site. Through simple random sampling, 15% of the sampled HWs selected for the knowledge assessment survey were surveyed for the practice improvement assessment. A total of 88 ANMs (45 from the intervention districts and 43 from the control districts) were surveyed during the baseline and endline.^18^ The net change obtained from the difference of means (Table 1) showed that significant changes were observed with ANMs properly performing duties, such as updating the maternal and child protection cards and counterfoils immediately after vaccination, asking caregivers to wait for 30 minutes after vaccination, and not using the vaccine after 4 hours of reconstitution. Likewise, improvement was seen in standard practices, such as placing heat-sensitive vaccines on ice packs and administering vaccines at the correct site on a child.

**TABLE 1.** Paired T-Test Results Showing Learning Effectiveness After RISE Training Indicative Through Practice Improvement Assessment in Intervention Districts in India

**Correct Practices Observed**	**No. (%) of ANMs Practicing Correctly Before RISE Training** **(N=45)**	**X**	**No. (%) of ANMs Practicing Correctly After RISE Training** **(N=45)**	**X**	***P*-Value**	**Net Change**
1. Mark date and time after opening each vaccine vial	25 (56)	1.6	41 (91)	1.9	<.001	0.356
2. Place heat-sensitive vaccines on ice pack	23 (51)	1.5	31 (69)	1.7	<.05	0.178
3. Does not use reconstituted vaccines after 4 hours of reconstitution	42 (93)	1.1	45 (100)	2.0	<.001	0.933
4. Does not touch needle during vaccination	41 (91)	2.5	44 (98)	2.4	NS	0.044
5. Use correct route of vaccination	35 (78)	2.2	42 (93)	2.4	NS	0.178
6. Uses correct site for administering vaccines to a child	38 (84)	2.3	44 (98)	2.5	<.001	0.156
7. Cut hub of both auto-disable and disposable syringes immediately after use	38 (84)	2.2	42 (93)	2.4	<.05	0.156
8. Uses Red bag for collecting the plastic part of the syringes immediately after using the hub cutter	33 (73)	1.9	40 (89)	2.1	NS	0.222
9. Update maternal and child protection card and counterfoil immediately after vaccination	10 (22)	0.6	39 (87)	1.9	<.001	1.289
10. All 4 key messages given to the caregivers	14 (31)	1.0	26 (58)	1.1	NS	0.089
11. Ask the caregivers to wait for 30 minutes after vaccination	10 (22)	0.7	35 (78)	1.5	<.001	0.800

Sample health workers (N=45) in the intervention districts.

Abbreviations: ANM, auxiliary nurse midwife; NS, not significant.

JSIPL conducted qualitative in-depth interviews with 30 HWs in intervention districts who completed the RISE modules, as well as 15 block and 5 district medical officers who were involved in implementing the application in their districts. All participants provided consent before being interviewed. Survey questions inquired about participants’ experiences attending the RISE training, taking the RISE modules on their mobile phones, benefits or downsides of the RISE app in their work, and complementarity to the traditional classroom-based instructor-led training.^18^

Based on the encouraging outcome of the pilot phase, showing HWs’ knowledge and skill improvements, the MOHFW decided to implement a phase-wise scale-up of RISE to impart routine immunization training to all vaccinators throughout India (Table 2). From November 1, 2021 to January 31, 2022, Phase 1 targeted 33 districts in 3 states (Himachal Pradesh, Madhya Pradesh, and Odisha), reaching more than 12,500 HWs. An additional 385 qualitative in-depth interviews were conducted throughout Phase 1 scale-up by a third-party agency to assess system acceptability and sustainability of the RISE app because this was not assessed in the pilot phase. During Phase 1 scale-up, interview respondents noted improvements in HWs’ performance after taking RISE modules.

**TABLE 2.** Phased Scale-up of RISE in India

**Phases**	**Target States**	**Target Districts**	**Target Learners**
Pilot Phase (2017–2020)	5	5	2,800+
Scale-up Phase 1 (2021–2022)	3	33	12,500+
Scale-up Phase 2 (2022–2024)	5	97	37,000+

*I have seen a lot of change after RISE, every time I go to a routine immunization session, I see that after RISE whatever the way of their speaking is, the way of making work plan, the method of vaccination has improved completely.* —Block medical officer, Himachal Pradesh

*HWs are gaining the knowledge through RISE training. As a result, we can rapidly provide better immunization services and effective communication with the community*. —Block program manager, Madhya Pradesh

From October 1, 2022 to June 30, 2024, Phase 2 scale-up is targeting more than 37,000 users in 98 districts of 5 states (Uttarakhand, Madhya Pradesh, Odisha, Tamil Nadu, and Maharashtra). Throughout this Phase, other feedback regarding the RISE modules and the app is being collected by the JSIPL State Teams via informal phone calls or WhatsApp messages from HWs.

## REACH AND IMPACT

Between March 1, 2020 and May 31, 2024, 39,839 learners in 136 districts of 6 states (Himachal Pradesh, Madhya Pradesh, Odisha, Maharashtra, Tamil Nadu, and Uttarakhand) registered and accessed the content on the RISE app (Figure 2).

**FIGURE 3:**
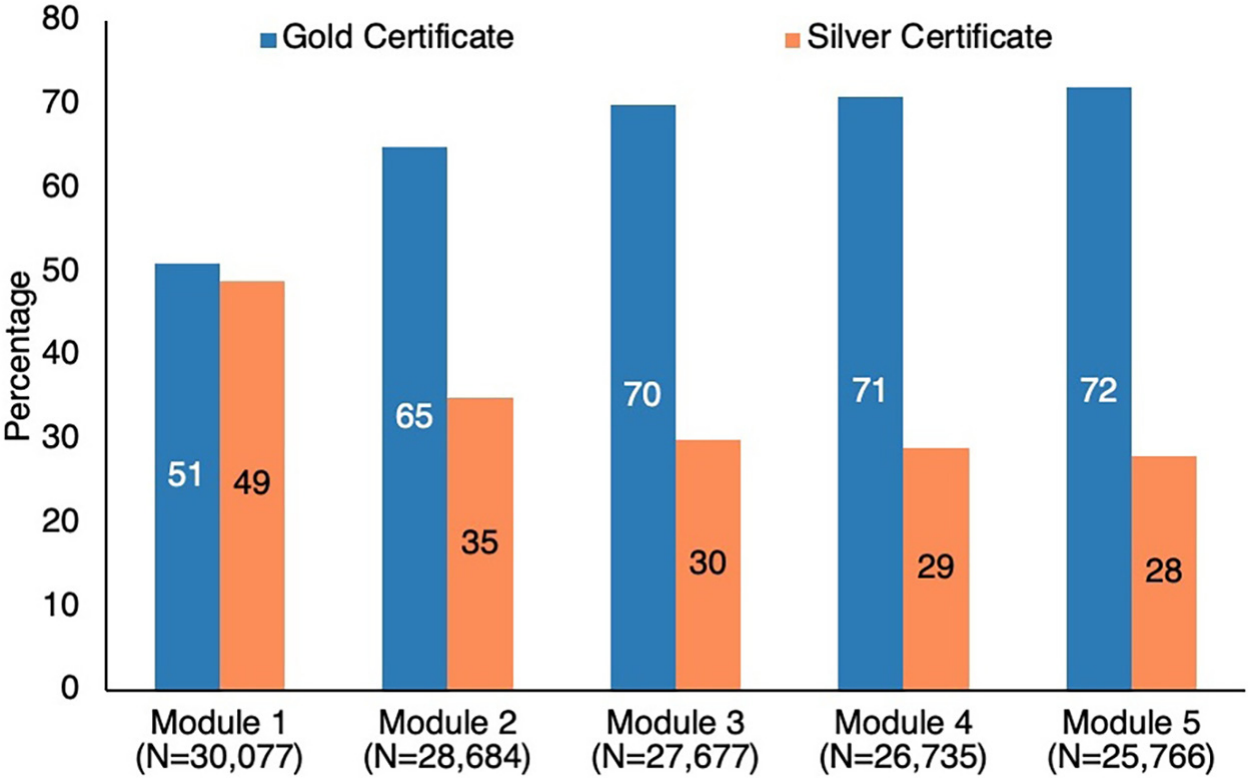
RISE App Learner’s Module Certificate Status, May 2024

As of May 31, 2024, approximately 25,766 learners have successfully completed all 5 training modules, amassing over 181,221 hours of logged training. A majority of learners have received gold certificates, the highest level of success (Figure 3). Around 65% of registered learners have completed all 5 modules. Reported challenges that require further attention are the app not working on select smartphones or ANMOL tablets, login errors, learners having to complete the module again if Internet connectivity dropped, or learners losing their progress if the language was switched mid-module.

**Figure:**
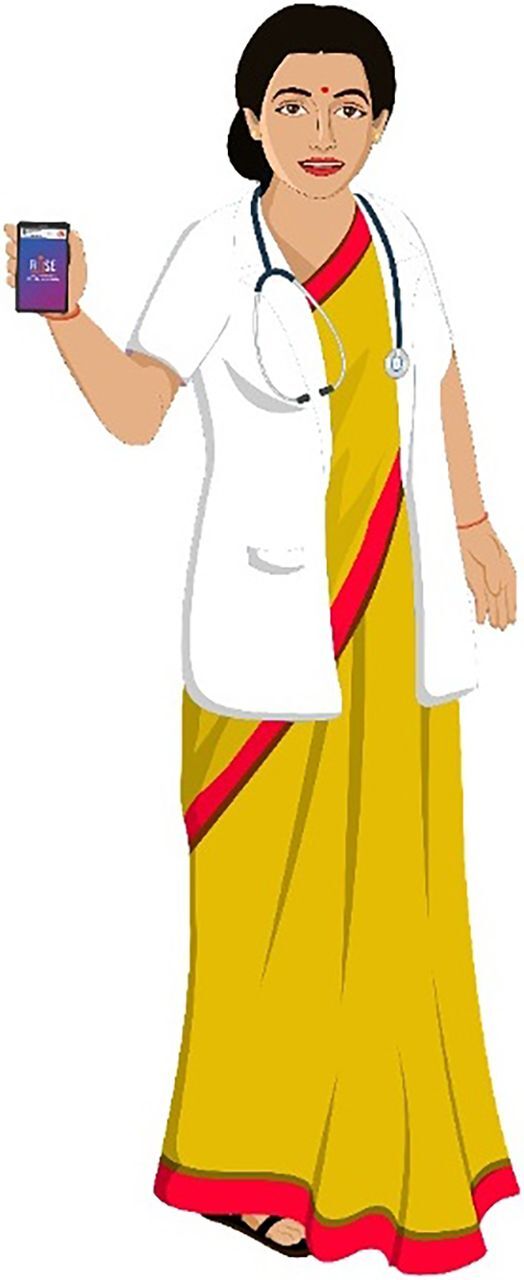
Narrator figure in RISE app. © John Snow India Private Limited

As of May 31, 2024, approximately 25,766 learners have successfully completed all 5 training modules, amassing over 181,221 hours of logged training.

JSIPL state teams, in consultation with government health officials, are working to use detailed dashboards to conduct virtual orientation sessions to help learners navigate the app and its limitations. Further work will need to be done on the app to overcome the limitations and provide accessibility to all HWs.

As per MOHFW guidance, RISE will be scaled up in 7 priority states (Bihar, Rajasthan, Uttar Pradesh, Meghalaya, Tamil Nadu, Maharashtra, and Arunachal Pradesh) across 236 districts and will ultimately result in the capacity-building of more than 110,000 HWs. The current phase is being implemented from December 31, 2023, to December 31, 2026, and will follow the same cascade approach as Phase 1 and Phase 2, in which the app will be launched at the state level, and district and block master trainers will be oriented and responsible for training the learners.

RISE has played a significant role in empowering women of all ages (as a majority of vaccinators are women) by helping them become more technologically adept.

*I am about to retire soon from the system. But there is no age to learning. I am excited to see that new innovative technology is coming into play to train the health workforce. I have come to take the training today and also was able to generate a certificate.* — Female HW, Maharashtra

*The app is made in such a way that even HWs from senior ages are also able to complete the modules without much support and supervision.* —Berasia block health officer, Madhya Pradesh

RISE is also easily scalable and could become less cost intensive with each added learner. The methodology can be replicated in other health areas and is likely a next step in India, as JSIPL has received feedback from various agencies and stakeholders asking for other health topics to be covered in the app.

## IMPLEMENTATION CHALLENGES AND SOLUTIONS

While deploying the RISE app, a few implementation challenges arose. Solutions were developed based on user feedback, observations, and interactions with state- and national-level stakeholders. Some challenges persist, and solutions are being worked on to mitigate future ones.

### Digital Literacy

Digital literacy was a challenge when working toward strengthening the HWs’ capacity on their smartphones. The 2018 capacity-building needs assessment findings showed that more than 80% of participants were already well-versed in the use of smartphones. To help close the gap in capacity, training tools, such as a demonstrative video and user manual that explained all RISE features and functions (e.g., how to download it and log in and how to access and complete modules), were provided during orientation. Peer-to-peer learning was also encouraged for any outstanding questions.

### Internet Connectivity

In the high terrain geography, many learners have low Internet access that leads to frequent drops of coverage from the app. To overcome this, learners were encouraged to access the modules in places where Internet connectivity was strong (e.g., their health facilities or homes). Despite this suggestion, many learners often needed better connectivity and submitted WhatsApp help requests about idling modules.

*Course is good but face a lot of problems because of its slow speed.* —HW

Continuous endeavors are being made to make the modules simple and clean by adding only essential blocks, leading to improved loading time. In 2023, version 2 of the modules was published with easier navigation to help learners progress smoothly.

### Orientation of State/District Supervisors on the App

A cascade training approach was adopted to orient supervisors on the app. In some states, orientation workshops took longer than expected due to competing priorities, and a lag existed before the block level received their orientations. However, continuous follow-up and advocacy by JSIPL to state/district health officials improved this issue with most supervisors. Orientations are now conducted within a reasonable time. It is essential to course correct delayed orientations, as supervisors were and will continue to be vital in registering HWs and collecting their information (e.g., names, phone numbers, work facility, and supervisors’ information). Supervisors consistently provide information on learners who have retired, transferred, or new learners who need to be registered. Supervisor’s support is key to increasing the learner base of RISE.

*I was given the special responsibility to ensure that all the workers are technically trained. Every week, progress from the dashboard was extracted from the software and shared at the block level under the guidance of the district immunization officer. Due to this, I used to personally contact those workers and motivate them to complete the module.* —Routine immunization data manager, Datia district

### Multilingual Translation

Language translation of the modules was a barrier to keeping the momentum of onboarding learners from one state to another state. Initially, the app was produced in English, but there was limited user understanding because many vaccinators at the block level are not English language learners. Therefore, for the pilot phase, the app was translated and produced in Hindi, Marathi, Odia, and Tamil to accommodate a broader learning group. In the next phase of the scale-up, the proposed targeted states will mainly be conversant in Hindi. Existing Hindi modules will be used and contain updated guidelines but will not require additional language translation. As the need for additional languages increases, the app will be translated into other languages.

### Learner Issues and Support

In 2020, when the RISE modules were piloted, some learners faced technology or app-related issues.

*Some workers could not complete and left the training in between because of experiencing many technical problems while using the RISE application.* —District supervisor

To ensure smooth learning by addressing learner issues, RISE team members were added to existing district- and block-level WhatsApp groups to assist learners with their queries and ensure no learners’ inquiries were overlooked and unanswered.

*I was unable to proceed at one screen and I posted the problem in WhatsApp group. My problem was sorted immediately with friendly guidance by pointing out one click button, which I had been missing to notice. —*Village health nurse, Acharapakkam Block, Tamil Nadu

Tools (e.g., chatbots, help centers, and help tickets) are being incorporated to further empower learners to find answers to popular questions independently.

## SUSTAINABILITY

Effective implementation and sustainability of a program require leadership, coordinated efforts from a team of skilled professionals, government acceptance, detailed planning and project management, ongoing stakeholder engagement, continuous curriculum content updating, and financial stability.

In the 6 states where the RISE app has been implemented, it is being accessed by more than 81% of enrolled learners.

*The best thing about the RISE app is that I could take the training course at any point in time and therefore can spare time for the aftercare of my baby. I could pause and play the course anytime and that’s what is the most attractive function of this training.* — HW, Madhya Pradesh

Learners appreciated the app’s features, such as instant recognition of completion achievement, holistic overview of the core areas of immunization, supportive supervision, peer-to-peer learning, and easy dissemination of upcoming changes in the guideline. Learners from various regions shared their testimonials in the dashboard and WhatsApp groups.

*RISE is a true companion; all the routine immunization-related knowledge had been refreshed through the app as most of the doubts have been cleared through the training.* —HW, Himachal Pradesh

An important aspect of sustainability is to keep content continuously updated. Given the high acceptance and use rates, the RISE app is being used for new vaccines being introduced and forthcoming changes in the guidelines of routine immunization. The JSIPL RISE team has proactively received updates from the Government of India and updated the learning modules in line with the new guidance. By engaging stakeholders and users, the team can ensure the app is relevant, useful, and acceptable to them.

Similarly, this platform can be leveraged and used for other cadres of national health programs. The app is on an open platform, and the software is compatible with other software used by the Government of India (e.g., NIC server, ANMOL platform). Hence, it can be used to disseminate training modules for capacity-building of other cadres, such as medical officers and accredited social health activists.

The RISE training has been integrated into other important trainings at the state level, such as the measles-rubella vaccine and Intensified Mission Indradhanush 5.0, to show vaccine administration techniques. The overarching goal is for RISE to become an intrinsic part of the routine immunization training methodology and be owned and run wholly by the MOHFW. The interoperability with existing platforms and low software cost increase the app’s sustainability.

The RISE program has received continuous support and ownership from states where it has been implemented. The constant communication with state-level stakeholders secured buy-in, created a sense of ownership of the app, and decreased resistance to its implementation. A prime example of state-level acceptance was seen when the Uttarakhand and Madhya Pradesh Joint Director of Health Services in UIP, also known as SEPIO, personally attended in-person and virtual RISE workshops, training, and orientations to HWs. RISE performance reviews have been integrated into monthly review meetings at the block and district levels and as a routine immunization agenda item at state- and national-level review meetings.

Gavi, the Vaccine Alliance, funded the pilot phase of the RISE app. However, to scale up, the Government of India took ownership and adopted a model of cost-sharing where the state governments partly covered the cost of the program through the National Health Mission and supplied manpower. JSIPL stores and monitors RISE data and activities but uses established review and feedback mechanisms at the state-, district-, and sub-district levels. Government officials, using insights from dashboards, often send letters instructing specific learners to complete their pending modules. They further use these data to identify low-performing districts and hold reorientations for HWs. Thus, the RISE scale-up reflects a public-private partnership model, with stewardship from the Government of India and technical assistance from JSIPL.

## CONCLUSION

RISE started as an explorative innovation to address the capacity-building gaps in India’s immunization workforce, and through an intentional and systematic process, it has come a long way to establish itself as a credible, scalable, and sustainable model of training. The application started with a coverage of approximately 2,800 learners in 5 districts and now caters to around 40,000 learners across 101 districts in 6 different states across India. The Government of India has already mandated that the RISE app be expanded in all districts of the country, with partial funding provided through the National Health Mission budgets, thus ensuring a step forward toward sustainability.

Feedback from the learners indicates satisfaction and appreciation for RISE, primarily due to its user accessibility, flexibility in time and place to complete modules, built-in interactive quizzes and games, and assessments paired with award certificates. Simultaneously, supervisors have commended RISE for not only improving the knowledge and skills of their workforce but also providing them with a structured monitoring and evaluation mechanism with a real-time live dashboard.

## Supplementary Material

GHSP-D-24-00006_supplements.pdf

## Notes

**Peer Reviewed**
