# Supplementary material for: Journey From a Digital Innovation to a Sustainable Health Worker Capacity-Building App in India: Experiences, Challenges, and Lessons Learned
Source: Glob Health Sci Pract. 2024 Jun 27;12(3):e2400006. doi: 10.9745/GHSP-D-24-00006 (PMC11216696; doi:10.9745/GHSP-D-24-00006)

Supplement to: Chase R, Sanyal S, Singh P, et al. Journey from a digital innovation to a sustainable health worker capacity-building app in India: experiences, challenges, and lessons learned. *Glob Health Sci Pract*. 2024;12(3):e2400006. https://doi.org/10.9745/GHSP-D-24-00006

Supplement 1. RISE LMS Application Back-end Technical Specifications:

- Link: https://risemohfw.in/
- LMS Platform used: Open edX
- Availability of the Application: Web, Android, and iOS
- Application Framework: JavaScript Object Notation
- Web framework: Django
- Server hosted on: Agency on premise Sever along with aws cloud server
- Security audits: Yes; September 2023-CERT-In Empanelled agency
- Cross-platform - mobile and web view
- Programming language: web-Python

Technical specifications for the iOS version of the application:

- App size: 61.9 MB
- Minimum iOS support: iOS 13.0
- Programming languages: Objective-C, Swift
- Development tool: Xcode

Technical specifications for the Android version of the application:

- App size: 49.03 MB
- Minimum Android version: Android 7.0 Nougat
- Programming languages: Java, Kotlin
- Development tool: Android Studio

Supplement to: Chase R, Sanyal S, Singh P, et al. Journey from a digital innovation to a sustainable health worker capacity-building app in India: experiences, challenges, and lessons learned. *Glob Health Sci Pract*. 2024;12(3):e2400006. https://doi.org/10.9745/GHSP-D-24-00006

Supplement 2. Knowledge Assessment Sampling Frame and Sample Size

Intervention Districts

**State**	**Intervention District**	**No. of Blocks**	**Sample Blocks**	**No. of ANMs**	**Sample size for ANM Knowledge Assessment**
Himachel Pradesh	Shimla	10	3	310	57
Madhya Pradesh	Bhopal	2	2	135	42
Maharashta	Pune	13	3	1334	69
Odisha	Khordha	10	3	254	54
Tamil Nada	Kancheepuram	13	3	257	54
	**Total**	**44**	**14**	**2290**	**276**

Control Districts

**State**	**Control District**	**No. of Blocks**	**Sample Blocks**	**No. of ANMs**	**Sample size for ANM Knowledge Assessment**
Himachel Pradesh	Solan	5	3	184	48
Madhya Pradesh	Indore	4	2	289	54
Maharashta	Nagpur	14	3	574	63
Odisha	Jharsuguda	5	3	90	36
Tamil Nada	Madurai	13	3	298	57
	**Total**	**41**	**14**	**1435**	**258**

Supplement to: Chase R, Sanyal S, Singh P, et al. Journey from a digital innovation to a sustainable health worker capacity-building app in India: experiences, challenges, and lessons learned. *Glob Health Sci Pract*. 2024;12(3):e2400006. https://doi.org/10.9745/GHSP-D-24-00006

Comparison of Baseline and Endline Assessment in RISE Intervention Districts

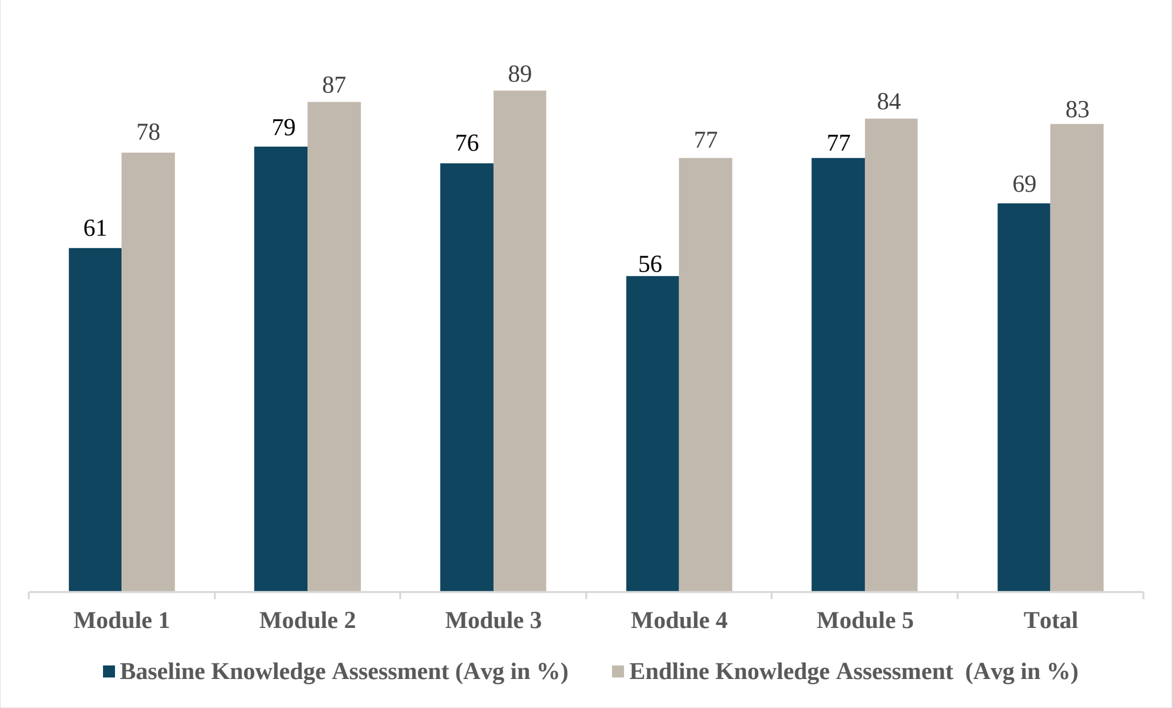

Comparison of Baseline and Endline Assessment in RISE Control Districts

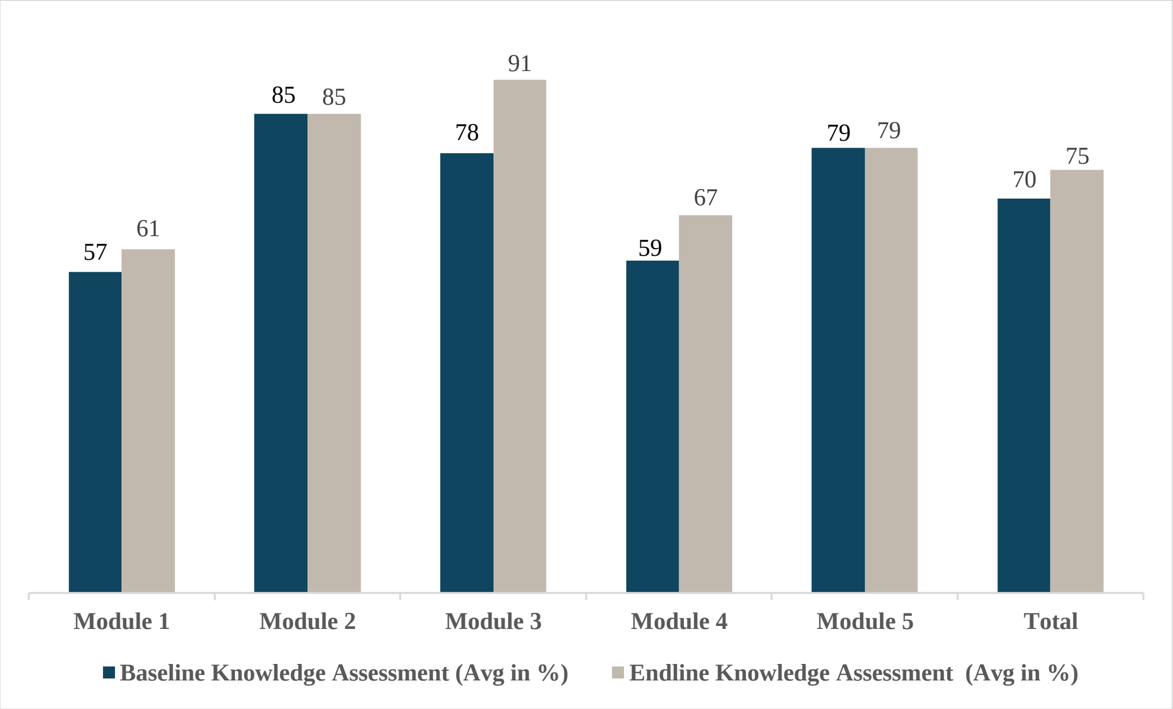

Supplement: GHSP-D-24-00006_supplements.pdf [file GHSP-D-24-00006_supplements.pdf]
